# Supplementary material for: Safety and clinical impact of adenosine stress perfusion cardiac magnetic resonance in Asian patients with known or suspected coronary artery disease
Source: PLoS One. 2023 Oct 12;18(10):e0292950. doi: 10.1371/journal.pone.0292950 (PMC10569532; doi:10.1371/journal.pone.0292950)

PLOS ONE

Safety and Clinical Impact of Adenosine Stress Perfusion Cardiac Magnetic Resonance in Asian Patients with Known or Suspected Coronary Artery Disease

--Manuscript Draft--

**Manuscript Number:**	PONE-D-23-23709
**Article Type:**	Research Article
**Full Title:**	Safety and Clinical Impact of Adenosine Stress Perfusion Cardiac Magnetic Resonance in Asian Patients with Known or Suspected Coronary Artery Disease
**Short Title:**	Safety and Clinical Impact of Adenosine Stress CMR
**Corresponding Author:**	Rungroj Krittayaphong<br>Mahidol University Faculty of Medicine Siriraj Hospital<br>Bangkok, THAILAND
**Keywords:**	Adenosine; Cardiac magnetic resonance; Impact; Safety; Stress test
**Abstract:**	Background: Adenosine stress cardiac magnetic resonance (CMR) imaging is increasingly utilized for evaluating patients with known or suspected coronary artery disease (CAD). This study aims to assess the safety and clinical impact of adenosine stress CMR in a tertiary care setting in Thailand.<br>Methods: A total of 3,768 consecutive patients aged 18 years and above who underwent adenosine stress CMR between 2017 and 2020 were included in the study. Patient records were reviewed to collect data on clinical characteristics, hemodynamic measurements, complications during or immediately after CMR, and the rates of clinical changes resulting from CMR.<br>Results: Among the included patients, the primary indications for adenosine stress CMR were risk stratification in suspected CAD (70.8%) and the assessment of myocardial ischemia/viability in patients with known CAD (26.5%). There were no reported deaths or acute myocardial infarctions during the procedure. Major complications, specifically acute pulmonary edema requiring hospital observation or admission for further management, occurred in four patients (0.11%), all of whom were elderly with a history of heart failure. Non-major complications were observed in 13.7% of patients, with dyspnea (9.8%) and mild chest pain (5.6%) being the most common. CMR provided a completely new diagnosis in 26.2% of patients. Overall, stress CMR resulted in a change in diagnosis or management for 48% of patients.<br>Conclusion: Adenosine stress CMR was found to be safe and had a significant impact on clinical management in Asian patients with known or suspected CAD. These findings support the use of adenosine stress CMR as a valuable tool for evaluating and guiding treatment decisions in this patient population.
**Order of Authors:**	Yodying Kaolawanich, MD
	Thammarak Songsangjinda, MD
	Ahthit Yindeengam, BSc
	Kanchalaporn Jirataiporn, BSN
	Rungroj Krittayaphong
**Additional Information:**	
**Question**	**Response**
**Financial Disclosure**<br><br>Enter a financial disclosure statement that describes the sources of funding for the work included in this submission. Review the submission guidelines for detailed requirements. View published research articles from *PLOS ONE* for specific	The author(s) received no specific funding for this work.

Powered by Editorial Manager® and ProduXion Manager® from Aries Systems Corporation

examples.<br><br>This statement is required for submission and **will appear in the published article** if the submission is accepted. Please make sure it is accurate.<br><br>**Unfunded studies**<br>Enter: *The author(s) received no specific funding for this work.*<br><br>**Funded studies**<br>Enter a statement with the following details:<br>• Initials of the authors who received each award<br>• Grant numbers awarded to each author<br>• The full name of each funder<br>• URL of each funder website<br>• Did the sponsors or funders play any role in the study design, data collection and analysis, decision to publish, or preparation of the manuscript?<br>• **NO** - Include this sentence at the end of your statement: *The funders had no role in study design, data collection and analysis, decision to publish, or preparation of the manuscript.*<br>• **YES** - Specify the role(s) played.<br><br>* typeset	
**Competing Interests**<br><br>Use the instructions below to enter a competing interest statement for this submission. On behalf of all authors, disclose any competing interests that could be perceived to bias this work—acknowledging all financial support and any other relevant financial or non-financial competing interests.<br><br>This statement is required for submission and **will appear in the published article** if the submission is accepted. Please make sure it is accurate and that any funding sources listed in your Funding Information later in the submission form are also declared in your Financial Disclosure statement.	The authors have declared that no competing interests exist.

Powered by Editorial Manager® and ProduXion Manager® from Aries Systems Corporation

View published research articles from *PLOS ONE* for specific examples.<br><br>**NO authors have competing interests**<br><br>Enter: *The authors have declared that no competing interests exist.*<br><br>**Authors with competing interests**<br><br>Enter competing interest details beginning with this statement:<br><br>*I have read the journal's policy and the authors of this manuscript have the following competing interests: [insert competing interests here]*<br><br>* typeset	
**Ethics Statement**<br><br>Enter an ethics statement for this submission. This statement is required if the study involved:<br><br>• Human participants<br>• Human specimens or tissue<br>• Vertebrate animals or cephalopods<br>• Vertebrate embryos or tissues<br>• Field research<br><br>Write "N/A" if the submission does not require an ethics statement.<br><br>General guidance is provided below. Consult the submission guidelines for detailed instructions. **Make sure that all information entered here is included in the Methods section of the manuscript.**	This study was approved by the Siriraj Institutional Review Board (SIRB), Faculty of Medicine Siriraj Hospital, Mahidol University. COA no. 225/2020. The need for consent was waived by the board due to its retrospective nature and as all personal identifying information was obliterated.

Powered by Editorial Manager® and ProduXion Manager® from Aries Systems Corporation

**Format for specific study types**<br><br>**Human Subject Research (involving human participants and/or tissue)**<br>• Give the name of the institutional review board or ethics committee that approved the study<br>• Include the approval number and/or a statement indicating approval of this research<br>• Indicate the form of consent obtained (written/oral) or the reason that consent was not obtained (e.g. the data were analyzed anonymously)<br><br>**Animal Research (involving vertebrate animals, embryos or tissues)**<br>• Provide the name of the Institutional Animal Care and Use Committee (IACUC) or other relevant ethics board that reviewed the study protocol, and indicate whether they approved this research or granted a formal waiver of ethical approval<br>• Include an approval number if one was obtained<br>• If the study involved *non-human primates*, add *additional details* about animal welfare and steps taken to ameliorate suffering<br>• If anesthesia, euthanasia, or any kind of animal sacrifice is part of the study, include briefly which substances and/or methods were applied<br><br>**Field Research**<br><br>Include the following details if this study involves the collection of plant, animal, or other materials from a natural setting:<br>• Field permit number<br>• Name of the institution or relevant body that granted permission	
**Data Availability**<br><br>Authors are required to make all data underlying the findings described fully available, without restriction, and from the time of publication. PLOS allows rare exceptions to address legal and ethical concerns. See the PLOS Data Policy and FAQ for detailed information.	Yes - all data are fully available without restriction

Powered by Editorial Manager® and ProduXion Manager® from Aries Systems Corporation

A Data Availability Statement describing where the data can be found is required at submission. Your answers to this question constitute the Data Availability Statement and **will be published in the article**, if accepted.<br><br>**Important:** Stating 'data available on request from the author' is not sufficient. If your data are only available upon request, select 'No' for the first question and explain your exceptional situation in the text box.<br><br>Do the authors confirm that all data underlying the findings described in their manuscript are fully available without restriction?	
**Describe where the data may be found in full sentences. If you are copying our sample text, replace any instances of XXX with the appropriate details.**<br><br>• If the data are **held or will be held in a public repository**, include URLs, accession numbers or DOIs. If this information will only be available after acceptance, indicate this by ticking the box below. For example: *All XXX files are available from the XXX database (accession number(s) XXX, XXX.).*<br>• If the data are all contained **within the manuscript and/or Supporting Information files**, enter the following: *All relevant data are within the manuscript and its Supporting Information files.*<br>• If neither of these applies but you are able to provide **details of access elsewhere**, with or without limitations, please do so. For example:<br><br>*Data cannot be shared publicly because of [XXX]. Data are available from the XXX Institutional Data Access / Ethics Committee (contact via XXX) for researchers who meet the criteria for access to confidential data.*<br><br>*The data underlying the results presented in the study are available from (include the name of the third party*	None

and contact information or URL).<br>• This text is appropriate if the data are owned by a third party and authors do not have permission to share the data.<br><br>* typeset	
Additional data availability information:	

Powered by Editorial Manager® and ProduXion Manager® from Aries Systems Corporation

Manuscript

Click here to access/download;Manuscript;Manuscript 26July2023.docx

Safety and Clinical Impact of Adenosine Stress Perfusion Cardiac Magnetic Resonance in Asian Patients with Known or Suspected Coronary Artery Disease

Yodying Kaolawanich, MD[1], Thammarak Songsangjinda, MD[1,2], Kanchalaporn Jirataiporn, BSN[1], Ahthit Yindeengam, BSc[1], Rungroj Krittayaphong, MD[1]

[1]Division of Cardiology, Department of Medicine, Faculty of Medicine Siriraj Hospital, Mahidol University Bangkok, Thailand

[2]Division of Cardiology, Department of Medicine, Faculty of Medicine, Prince of Songkla University, Songkhla, Thailand

Running title: Safety and Clinical Impact of Adenosine Stress CMR

Abstract word count: 261

Manuscript word count: 3,355

Tables: 4

Figures: 3

Correspondence to:

Rungroj Krittayaphong, MD

Division of Cardiology, Department of Medicine

Faculty of Medicine Siriraj Hospital, Mahidol University

2 Wanglang Road, Bangkok Noi

Bangkok 10700, Thailand

Tel: (+66) 2-419-6104; Fax: (+66) 2-412-7412

E-mail: rungroj.kri@mahidol.ac.th

Abstract

Background: Adenosine stress cardiac magnetic resonance (CMR) imaging is increasingly utilized for evaluating patients with known or suspected coronary artery disease (CAD). This study aims to assess the safety and clinical impact of adenosine stress CMR in a tertiary care setting in Thailand.

Methods: A total of 3,768 consecutive patients aged 18 years and above who underwent adenosine stress CMR between 2017 and 2020 were included in the study. Patient records were reviewed to collect data on clinical characteristics, hemodynamic measurements, complications during or immediately after CMR, and the rates of clinical changes resulting from CMR.

Results: Among the included patients, the primary indications for adenosine stress CMR were risk stratification in suspected CAD (70.8%) and the assessment of myocardial ischemia/viability in patients with known CAD (26.5%). There were no reported deaths or acute myocardial infarctions during the procedure. Major complications, specifically acute pulmonary edema requiring hospital observation or admission for further management, occurred in four patients (0.11%), all of whom were elderly with a history of heart failure. Non-major complications were observed in 13.7% of patients, with dyspnea (9.8%) and mild chest pain (5.6%) being the most common. CMR provided a completely new diagnosis in 26.2% of patients. Overall, stress CMR resulted in a change in diagnosis or management for 48% of patients.

Conclusion: Adenosine stress CMR was found to be safe and had a significant impact on clinical management in Asian patients with known or suspected CAD. These findings support the use of adenosine stress CMR as a valuable tool for evaluating and guiding treatment decisions in this patient population.

Keywords: Adenosine; Cardiac magnetic resonance; Impact; Safety; Stress test.

Introduction

Coronary artery disease (CAD) is a significant global health burden, with a high prevalence and impact on individuals and healthcare systems. Accurate diagnosis and risk stratification of patients with known or suspected CAD are of utmost importance. Stress cardiac magnetic resonance (CMR), commonly using adenosine, is increasingly being utilized. CMR provides a comprehensive assessment of CAD with very high accuracy.(1) It can assess global and regional ventricular function, myocardial ischemia, and infarction in a single study. Moreover, stress CMR offers strong evidence for prognosis, including mortality, in patients with known or suspected CAD.(2, 3)

Stress CMR has demonstrated a significant impact on diagnosis and patient management. Previous studies have reported that stress CMR resulted in a substantial alteration in clinical care for approximately 60-70% of patients.(4, 5) The safety profile of stress CMR has been documented in prior studies. Menadas JV et al. demonstrated that dipyridamole stress CMR was feasible and safe, with a rate of severe immediate complications of 0.06%.(6) Bruder et al. also showed a very low rate of severe complications of stress CMR using adenosine and dobutamine, with a rate of 0.026%.(4) However, there are limited studies regarding the clinical impact and safety of stress CMR in Asia, and most of them were relatively small in scale.(7, 8)

This study aims to assess the safety and clinical impact of adenosine stress CMR in a tertiary care setting in Thailand.

Methods

Study Population

This was a retrospective observational study conducted at Siriraj Hospital, an academic medical center in Bangkok, Thailand. We included consecutive patients aged 18 years or older who were referred for adenosine stress CMR for clinical purposes during 2017 to 2019. Patients were excluded if they had incomplete CMR scans or if they did not have follow-up data after CMR. Information on baseline demographic variables was obtained from the electronic medical record. Hypertension was defined as a self-reported history of hypertension, the use of antihypertensive medication, or an office blood pressure of ≥140/90 mmHg. Diabetes was defined as a self-reported history of diabetes and/or receiving anti-diabetic treatment, or a fasting glucose level of ≥126 mg/dL. Dyslipidemia was defined as a total cholesterol level of ≥240 mg/dL, a low-density lipoprotein (LDL) cholesterol level of ≥130 mg/dL, a high-density lipoprotein (HDL) cholesterol level of <40 mg/dL, a triglyceride level of ≥200 mg/dL, and/or treatment with a lipid-lowering agent. The protocol for this study was approved by the Siriraj Institutional Review Board. The Ethics Committee waived the requirement of written informed consent for participation due to the retrospective design of the study. Data were accessed for research purposes between 1st April 2020 and 30th January 2023.

CMR Protocol and Analysis

CMR was performed using standardized protocols recommended by the Society of Cardiovascular Magnetic Resonance (SCMR)(9) on a 3T scanner (Philips Medical Systems, Best, The Netherlands) and interpreted by experienced readers. The CMR protocol included ventricular function assessment using a standard steady-state free precession sequence obtained in short and long-axis views, myocardial first-pass perfusion, and late gadolinium enhancement (LGE). Three

short-axis slices at the apical, mid, and basal left ventricular levels were chosen for perfusion imaging with an electrocardiographic (ECG)-triggered, T1-weighted, inversion recovery single-shot turbo gradient echo sequence. Typical image parameters were described elsewhere.(10)

Venous access was obtained in both upper limbs, with one being used for continuous adenosine infusion and the other being used for gadolinium contrast infusion at peak stress. Patients were continuously monitored with peripheral oxygen saturation probe, heart rate, and real- time electrocardiography (ECG) throughout the CMR scan. Blood pressure (BP) was recorded before starting the infusion and was checked every minute during adenosine infusion. The myocardial first-pass perfusion study was performed by injecting 0.05 mmol/kg of gadolinium contrast agent (Magnevist, Bayer Schering Pharma, Berlin, Germany) at a rate of 4 mL/s immediately after a 4-minute infusion of 140 mcg/kg/min of adenosine.(9) Patients were asked about any symptoms experienced during the infusion in order to assess their hemodynamic response and monitor for any potential complications. The adenosine infusion was discontinued prematurely if requested by the patient or in the presence of progressive or severe angina, dyspnea, a decrease in systolic pressure >40 mmHg, severe arrhythmias, or other adverse effects. LGE imaging obtained 10-15 minutes after administration of intravenous gadolinium (0.15 mmol/kg), as per published guidelines.(9) CMR images were interpreted by standard methods.(11) In brief, perfusion images were read, and each of the 16 segments was visualized (segment-17 at the apex was not visualized). Inducible ischemia was defined as a subendocardial perfusion defect that (i) persisted beyond peak myocardial enhancement and for several RR intervals, (ii) was more than two pixels wide, (iii) followed one or more coronary arteries, and (iv) showed absence of LGE in the same segment. Dark-banding artefacts were recorded if an endocardial dark band appeared at the arrival of contrast in the LV cavity before contrast arrival in the myocardium. LGE images

were also analyzed using visual assessment. LGE was considered present only if confirmed on both the short-axis and at least one other orthogonal plane. CMR diagnosis of CAD includes either a stress-inducible perfusion defect or the presence of ischemic LGE. The CMR diagnosis of nonischemic cardiomyopathy includes a nonischemic LGE pattern (e.g., midwall LGE for dilated cardiomyopathy) without stress-induced perfusion defects.

Complications of CMR

Major complications were defined as death, resuscitation, or any other condition related to the CMR procedure that required monitoring as an inpatient for at least 1 night after the CMR scan (e.g., acute myocardial infraction, acute pulmonary edema, ischemic stroke, arrhythmias, and so on). Non-major complications were defined as any complications related to CMR that did not fulfill the criteria for severe complications (e.g., dyspnea, chest pain, allergic reactions without shock, problems related to intravenous lines, and so forth).

Influence of CMR on Subsequent Clinical Management

Two cardiologists reviewed all clinical information dated before the CMR stress test. These reviewers were blinded to the results of the CMR and to the clinical course subsequent to the test. Two cardiologists independently assessed the clinical impact of each stress CMR by review of the electronic medical records through to the next outpatient visit with the ordering provider. "Completely new diagnosis" was defined as a new diagnosis occurring only if it was previously unknown by the referring physician.

Statistical Analysis

Statistical analyses were performed using IBM SPSS Statistics for Windows, version 20.0 (IBM Corp., Armonk, NY, USA). Continuous variables with a normal distribution were presented as mean ± standard deviation (SD), and continuous variables with a non-normal distribution were

presented as median and interquartile range. The normality distribution of the variables was examined using the Kolmogorov-Smirnov test. Categorical variables were presented as absolute numbers and percentages. Differences were compared using Student's unpaired t-test or Mann-Whitney U test for continuous variables, and the chi-square test or Fisher's exact test for categorical variables, as appropriate. All statistical tests were two-tailed, and p-values less than 0.05 were considered to indicate statistical significance.

Results

Patient Characteristics

A total of 3,785 patients were scanned within a period of 31 months. Flow diagram is presented in **Figure 1**. Ten patients with a contraindication to adenosine stress CMR (e.g., severe claustrophobia, known or suspected bronchoconstrictive or bronchospastic disease) and 7 patients with incomplete CMR scans were excluded. As a result, 3,768 patients were included in the final analysis. The main indications for adenosine stress CMR were risk stratification in suspected CAD (70.8%) and the assessment of myocardial ischemia/viability in patients with known CAD (26.5%) **(Figure 1)**.

Clinical characteristics of the patients are presented in **Table 1**. The mean age was 67.5±13.6 years, and 1,818 (48.2%) were male. Hypertension was the most prevalent CAD risk factor, observed in 73.9% of the patients. A total of 1,448 (38.4%) had diabetes mellitus. Among the patients, 1,082 had known CAD, with 264 having a prior myocardial infarction (MI). Forty-six percent of the patients presented with dyspnea, while twenty-two percent presented with chest pain.

Hemodynamic Measurements

An increase in heart rate was observed in 2,973 (79.0%) patients, and a reduction in blood pressure was observed in 2,987 (79.5%) patients. **Figure 2** depicts the hemodynamic effects of adenosine in our patients. Overall, there was a significant decrease in mean systolic and diastolic blood pressure (systolic: 135.2 ± 19.9 [rest] versus 124.8 ± 19.4 mmHg [during adenosine infusion], p < 0.001; diastolic: 74.7 ± 13.6 [rest] versus 66.0 ± 12.7 mmHg [during adenosine infusion], p < 0.001, respectively), accompanied by a compensatory increase in mean heart rate (75.7 ± 13.9 [rest] versus 83.4 ± 14.3 beats per minute [during adenosine infusion], p < 0.001).

Major and Non-major Complications

Major complications occurred in four patients (0.11%), all of whom had acute pulmonary edema requiring hospital observation or admission for further management. All four patients required hospital observation or admission for further management. All four patients were elderly (> 75 years old) and had previously been diagnosed with heart failure, classified as New York Heart Association (NYHA) class II-III (two with reduced left ventricular ejection fraction and two with preserved left ventricular ejection fraction). **Table 2** shows major complications during or immediately after CMR scans. There were no reported deaths or acute MI during the procedure.

Non-major complications occurred in 517 patients (13.7%), with 368 experiencing dyspnea (9.8%) and 209 reporting mild chest pain (5.6%) as the most common symptoms. The remaining patients experienced nausea (n=7), local complications at intravenous access sites (n=7), and contrast allergy without shock (n=3). No patients exhibited atrioventricular block. Among patients with dyspnea, there was no significant difference between those with a history of heart failure (n=34/447; 7.6%) and those without (n=334/3321; 10.1%), p=0.10. Similarly, among patients with mild chest pain, there was no significant difference between those with known CAD (n=65/1082; 6.0%) and those with suspected CAD (n=114/2686; 5.4%), p=0.43. Noted, there is no difference

in the rate of patients with positive stress CMR who developed dyspnea or mild chest pain during CMR ($p>0.05$ for both). **Figure 3** demonstrates non-major complications during or immediately after CMR scans.

Clinical Impact of CMR

Table 3 demonstrates the number of patients with a completely new diagnosis and changes in management after CMR. Adenosine stress CMR provided a diagnosis of 650 cases of CAD in patients with unknown CAD (before CMR). Additionally, comprehensive CMR examinations enabled the identification of nonischemic cardiomyopathies, such as dilated cardiomyopathy, hypertrophic cardiomyopathy, and myocarditis. CMR also facilitated the diagnosis of severe valvular disease, necessitating valvular surgery or intervention.

CMR resulted in therapeutic consequences in 1,110 (29.5%) patients, including changes in medication in 21.4% and invasive procedures in 14.2%. Overall, CMR had an impact on the diagnosis and patient management (completely new diagnosis and/or therapeutic consequences) in 48% of the patients.

Discussion

In this study, we have shown the main following findings: 1) Adenosine stress CMR was found to be safe, with a very low rate of major complications, specifically acute pulmonary edema (0.11%). All cases of acute pulmonary edema occurred in elderly patients with a history of heart failure. 2) Non-major complications were observed in 13.6% of the patients, with the most common being dyspnea and mild chest pain. These symptoms were not associated with patient characteristics such as heart failure or CAD status. 3) Adenosine stress CMR had a significant

impact on diagnosis and clinical management, resulting in 48% of patients receiving a new diagnosis or experiencing changes in their management.

Stress CMR has emerged as a prominent imaging modality for the detection and risk stratification of patients with known or suspected CAD. CMR can provide integrated information of cardiac function, structural changes such as LGE, and myocardial ischemia by studying myocardial perfusion in one examination.(12) Stress CMR, whether using vasodilators or dobutamine, has strong evidence for the diagnosis and prognosis of patients with known or suspected CAD.(1-3) Dipyridamole, one of the vasodilators used for stress CMR, has been shown to be safe. Monmeneu Menadas et al. reported a very low rate of severe immediate complications and demonstrated that inducible ischemia was the only factor identified as being associated with serious complications.(6) Adenosine has been the most commonly used vasodilator for stress CMR and has been shown to exhibit better sensitivity and specificity compared to dipyridamole.(13) Adenosine stress CMR has also demontrated a favorable safety profile in several studies conducted in Western countries.(4, 14)

The safety of adenosine stress CMR in Asia has been studied by Raj et al.(8) in 1,057 patients and Tsang et al.(7) in 98 patients. Both studies demonstrated a very low rate (<0.5%) of adverse events during or immediately after CMR. Our study, conducted with a larger patient population of 3,768 patients, also yielded consistent results. The majority of our patients exhibited a hemodynamic response to adenosine, characterized by a decrease in blood pressure and an increase in heart rate. There were no reported deaths or acute MIs during or immediately after CMR. Four patients with acute pulmonary edema were reported, all of whom were elderly with a history of heart failure. A study by Raj et al. in India demonstrated that three patients experienced

severe breathlessness during adenosine infusion and required further management, and one of them had a reduced LVEF.(8) This was consistent with our results.

The studies by Bruder and Menadas from Western countries showed differences compared to our data.(4, 6) Bruder et al. reported a very low rate of severe complications (0.026%) from the EuroCMR registry, independent of patient gender or age.(4) Similarly, Menadas et al. reported a very low rate of severe complications (0.08%), with no association found between complications and demographic, clinical, hemodynamic, or CMR-derived parameters.(6) Although our results differed from those of Bruder and Menadas, these discrepancies could be due to chance given the very low event rate. It's worth noting that our study included older patients with a higher prevalence of heart failure, more than double that of Menadas. Non-major complications occurred in 13.7% of our patients, with the most common being dyspnea and mild chest pain. These two symptoms were not related to patient characteristics such as heart failure symptom or CAD status. It is likely that they were effects of adenosine itself, which were not significant and could self-recover without requiring any specific treatment. Overall, we believe that adenosine stress CMR is safe for the majority of referred patients.

Several studies have reported a significant clinical impact of stress CMR. In our study, 48% of patients had a management impact, resulting in new diagnoses or changes in treatment based on CMR results. Approximately 10% of these patients had non-CAD diagnoses such as dilated cardiomyopathy, hypertrophic cardiomyopathy, or severe valvular disease, requiring different treatments including surgery or interventional procedures. These findings underscore the value of stress CMR. It is important to note that our results may differ from previous studies due to variations in endpoint definitions. For instance, McGraw et al. reported that stress CMR led to active changes in clinical care in about 70% of patients.(5) However, their study included

subspecialty consultations, preoperative clearances, or discharge from the Cardiology Clinic as criteria for active clinical change. On the other hand, Bruder et al., whose definition was more similar to ours, found that approximately 60% of patients had an impact from CMR results.(4) It is worth mentioning that Bruder's study included both stress and non-stress CMR. Nonetheless, stress CMR continues to have a significant impact on clinical decision-making, as observed in our study. **Table 4** presents a summary of patients' profiles, complications, and the clinical impact of stress CMR in our study, compared with previously published data.(4-8) All the studies reported the safety of stress CMR, and it was found to have a significant impact on patient management. It should be noted, however, that there was relatively limited data available from Asia.

Our study had several limitations. Firstly, it was conducted in a single tertiary center in Thailand, which may limit the generalizability of our findings to different regions. Additionally, the specific definition used in our study may differ from that of others, which could impact the comparability of results. However, our study contributes valuable data from an Asian country, which was previously scarce in this field. Secondly, certain complications such as bradyarrhythmia, atrioventricular block, or non-sustained ventricular tachycardia might not have been accurately recorded since we were unable to obtain 12-lead ECG recordings during CMR. However, this is a common limitation in many CMR studies, and it's important to note that no patients in our study required treatment for any arrhythmic events. Thirdly, due to the very low rate of events, we were unable to provide a reliable predictor for major complications. The limited number of events hindered our ability to perform a thorough and accurate analysis in this regard.

Conclusions

This study, the largest in Asia to date, aimed to demonstrate the safety profiles and clinical impact of adenosine stress CMR in Asian patients with known or suspected CAD. The findings of this study confirm that adenosine stress CMR is not only safe but also has a significant impact on clinical management in this patient population. These results provide strong support for the utilization of adenosine stress CMR as a valuable tool for evaluating and guiding treatment decisions in patients with known or suspected CAD.

Table 1. Clinical characteristics of the study population.

Characteristics	Total (n=3,768)
Age (years)	67.5±13.6
Male	1,818 (48.2)
Body mass index (kg/m^2)	25.5±4.6
CAD risk factors	
Hypertension	2,785 (73.9)
Hyperlipidemia	2,401 (63.7)
Diabetes mellitus	1,448 (38.4)
Cigarette smoking	115 (3.1)
Family history of CAD	23 (0.6)
Medical history	
Known CAD	1,082 (28.7)
Prior myocardial infarction	264 (7.0)
Revascularization, PCI	469 (12.4)
Revascularization, CABG	256 (6.8)
History of heart failure	447 (11.9)
Atrial fibrillation	392 (10.4)
Stroke	251 (6.7)
Symptoms	
Chest pain	832 (22.1)
Dyspnea	1,755 (46.6)
Other symptoms	24 (0.6)
No symptom	296 (7.9)
Medications	
Antiplatelet	2,025 (53.7)
Anticoagulant	303 (8.0)
ACEI or ARB	1,480 (39.3)
Beta blocker	1,977 (52.5)
Calcium channel blocker	1,100 (29.2)
Diuretic	744 (19.7)
Nitrate	746 (19.8)
Oral hypoglycemic agent	891 (23.6)
Insulin	204 (5.4)

Values are mean ± standard deviation or number (%).

Abbreviations: ACEI=angiotensin-converting enzyme inhibitors, ARB=angiotensin receptor blockers, CABG= coronary artery bypass graft, CAD=coronary artery disease, PCI=percutaneous coronary intervention.

Table 2. Major complications during or immediately after CMR scans.

	Total (n=3,768)
Major complications	4 (0.11)
Acute pulmonary edema	4 (0.11)
Acute myocardial infraction	0
[a]Unstable angina	0
Cardiac arrest	0
Sustained ventricular tachycardia	0
Ischemic stroke or transient ischemic attack	0
Severe symptomatic hypotension	0
Anaphylactic shock	0

Values are number (%).

Abbreviations: CMR=cardiac magnetic resonance.

[a]Chest pain of >20 min duration despite treatment, requiring hospital admission.

Table 3. Impact of CMR on diagnosis and patient management.

Variables	Total (n=3,768)
Completely new diagnosis	986 (26.2%)
CAD	650
Non-CAD	336
Dilated cardiomyopathy	93
Hypertrophic cardiomyopathy	55
Significant valvular disease	53
Vascular disease	35
Hypertensive heart disease	19
Myocarditis	12
Noncompaction cardiomyopathy	11
Others	58
Therapeutic consequences	1,110 (29.5%)
Change in medication	807
Add new medication	459
Aspirin	147
Clopidogrel	127
Anticoagulation	31
Beta blocker	147
Calcium channel blocker	43
Nitrate	88
Statin	100
Discontinued Medication	201
Dose changed	249
Invasive procedure	534
Impact on patient management (completely new diagnosis and/or therapeutic consequences)	1,814 (48.1%)

Values are number (%).

Abbreviations: CAD=coronary artery disease.

Table 4 Summary of patients' characteristics, complications, and clinical impact of stress CMR in published articles.

	Kaolawanich, et al.	Menadas, et al.(6)	Bruder, et al.(4)	McGraw, et al.(5)	Raj, et al.(8)	Tsang, et al.(7)
Year of enrollment	2017-2020	2004-2014	Until 2012	N/A	2018-2019	2013
Number of patients	3,768	11,984	27,781	350	1,057	98
Country	Thailand	Spain	Europe	USA	India	China
Asian	100%	0%	0%	0%	100%	100%
% with stress test	100%	100%	37.4%	100%	100%	100%
Stressors	Adenosine	- Dipyridamole (95.4%)<br>- Dobutamine (4.6%)	- Adenosine (78.3%)<br>- Dobutamine (21.7%)	Regadenoson	Adenosine	Adenosine
Age (mean)	67.5±13.6	64±12	60	59±13.7	55.5±9.9	64.0±11.4
Male	48.2%	60.4%	65.5%	46.3%	87.6%	71.4%
Hypertension	73.9%	64.2%	N/A	74.9%	N/A	N/A
Hyperlipidemia	63.7%	53.1%	N/A	53.4%	N/A	N/A
Diabetes mellitus	38.4%	26.2%	N/A	34.9%	N/A	N/A
Cigarette smoking	3.1%	18.0%	N/A	18.9%	N/A	N/A
Family history of CAD	0.6%	6.8%	N/A	N/A	N/A	N/A
Known CAD	28.7%	29.3%	N/A	31.4%	94.8%	52.0%
History of MI	7.0%	17.1%	N/A	N/A	N/A	N/A
History of heart failure	11.9%	4.9%	N/A	N/A	N/A	N/A
Most common CMR indication	Risk stratification in suspected CAD (70.8%)	N/A	Risk stratification in suspected CAD/Ischemia in known CAD (34.2%)	N/A	Known CAD	Risk stratification in suspected CAD (52.0%)
Complications during or immediately after stress CMR						
Death or acute MI	0	0	0	N/A	0	0
Major complication	4 (0.11%)<br>- Acute pulmonary edema (n=4)	10 (0.08%)<br>- Unstable angina (n=2)<br>- Acute pulmonary edema (n=2)	7 (0.07%)<br>- Non-sustained VT (n = 2)<br>- Ventricular fibrillation (n = 1)<br>- Overt heart failure (n = 2)	N/A	3 (0.28%)<br>- Unstable angina required hospital admission (n=3)	0

		- Sustained VT (n=1)<br>- Persistent AF (n=2)<br>- Asystole (n-1)<br>- TIA (n=1)<br>- Anaphylactic shock (n=1)	- Unstable angina (n = 1)<br>- Anaphylactic shock (n = 1)			
Non-major complications/symptoms	Non-major complications 13.7%	- Non-major complications 1.5%<br>- Minor symptoms 24.8%	Mild complications 3.6%	N/A	- Transient hypotension 1.8%<br>- Severe chest pain 0.5%<br>- Severe breathlessness 0.9%	Adverse effects 63.3%
Clinical impact of stress CMR						
New/change diagnosis	26.2%	N/A	8.1%[b]	N/A	N/A	N/A
Therapeutic consequences<br>- Change in medication<br>- Invasive procedure	 21.4%<br>14.2%	 N/A<br>N/A	 24.3%<br>23.1%	 18.3%<br>13.1%[c]	 N/A<br>N/A	 N/A<br>N/A
Impact on patient management[a]	48.1%	N/A	71.4%	69.5% [d]	N/A	N/A

[a]Diagnosis and/or therapeutic consequences.
[b]Completely new diagnosis not suspected before.
[c]Angiography with and without revascularization.
[d]Active change in clinical care.

Abbreviations: AF=atrial fibrillation, CAD=coronary artery disease, CMR=cardiac magnetic resonance, MI=myocardial infarction, VT=ventricular tachycardia, TIA=transiebnt ischemic attack.

Figure Captions

Figure 1. Flow diagram of the study.

Abbreviations: CAD=coronary artery disease, CMR=cardiac magnetic resonance.

Figure 2. Hemodynamic parameters at rest and during adenosine infusion.

Abbreviations: DBP=diastolic blood pressure, HR=heart rate, SBP=systolic blood pressure**.**

Figure 3. Non-major complications during or immediately after CMR scans.

List of abbreviations

CAD = coronary artery disease

CMR = cardiac magnetic resonance

ECG = electrocardiography

LGE = late gadolinium enhancement

MI = myocardial infarction

NYHA = New York Heart Association

SD = standard deviation

Acknowledgements: None.

Author contributions:

Concept and design, data acquisition, interpretation of data, manuscript preparation, manuscript revision, and manuscript review by YK, TS, and RK. Data acquisition, data analysis, manuscript revision, and manuscript review by YK, TS, KJ, and AY. All authors read, approved the final manuscript, and approved the submission of this manuscript for journal publication.

Funding: None.

Availability of data and materials

The datasets analyzed during the current study available from the corresponding author on reasonable request.

Declarations

Ethics approval and consent to participate

The protocol for this study was approved by the Siriraj Institutional Review Board. The Ethics Committee waived the requirement of written informed consent for participation due to the retrospective design of the study.

Consent for publication: Not applicable.

Competing interests: None.

References

1. Greenwood JP, Younger JF, Ridgway JP, Sivananthan MU, Ball SG, Plein S. Safety and diagnostic accuracy of stress cardiac magnetic resonance imaging vs exercise tolerance testing early after acute ST elevation myocardial infarction. Heart. 2007;93(11):1363-8.
2. Lipinski MJ, McVey CM, Berger JS, Kramer CM, Salerno M. Prognostic value of stress cardiac magnetic resonance imaging in patients with known or suspected coronary artery disease: a systematic review and meta-analysis. Journal of the American College of Cardiology. 2013;62(9):826-38.
3. Jahnke C, Nagel E, Gebker R, Kokocinski T, Kelle S, Manka R, et al. Prognostic value of cardiac magnetic resonance stress tests: adenosine stress perfusion and dobutamine stress wall motion imaging. Circulation. 2007;115(13):1769-76.
4. Bruder O, Wagner A, Lombardi M, Schwitter J, van Rossum A, Pilz G, et al. European cardiovascular magnetic resonance (EuroCMR) registry – multi national results from 57 centers in 15 countries. Journal of Cardiovascular Magnetic Resonance. 2013;15(1):9.
5. McGraw S, Romano S, Jue J, Bauml MA, Chung J, Farzaneh-Far A. Impact of Stress Cardiac Magnetic Resonance Imaging on Clinical Care. Am J Cardiol. 2016;118(6):924-9.
6. Monmeneu Menadas JV, Lopez-Lereu MP, Estornell Erill J, Garcia Gonzalez P, Igual Muñoz B, Maceira Gonzalez A. Pharmacological stress cardiovascular magnetic resonance: feasibility and safety in a large multicentre prospective registry. European Heart Journal - Cardiovascular Imaging. 2015;17(3):308-15.
7. Tsang KH, Chan WS, Shiu CK, Chan MK. The safety and tolerability of adenosine as a pharmacological stressor in stress perfusion cardiac magnetic resonance imaging in the Chinese population. Hong Kong Med J. 2015;21(6):524-7.
8. Raj V, Pudhiavan A, Hrishikesh VJ, Ali A, Kothari R. Safety profile of adenosine stress cardiac MRI in a tertiary hospital in India. Indian J Radiol Imaging. 2020;30(4):459-64.
9. Kramer CM, Barkhausen J, Bucciarelli-Ducci C, Flamm SD, Kim RJ, Nagel E. Standardized cardiovascular magnetic resonance imaging (CMR) protocols: 2020 update. Journal of Cardiovascular Magnetic Resonance. 2020;22(1):17.
10. Kaolawanich Y, Thongsongsang R, Songsangjinda T, Boonyasirinant T. Clinical values of resting electrocardiography in patients with known or suspected chronic coronary artery disease: a stress perfusion cardiac MRI study. BMC Cardiovascular Disorders. 2021;21(1).
11. Schulz-Menger J, Bluemke DA, Bremerich J, Flamm SD, Fogel MA, Friedrich MG, et al. Standardized image interpretation and post-processing in cardiovascular magnetic resonance - 2020 update. Journal of Cardiovascular Magnetic Resonance. 2020;22(1):19.
12. Wu KC. Variation on a theme: CMR as the "one-stop shop" for risk stratification after infarction? JACC Cardiovasc Imaging. 2009;2(7):843-5.
13. Hamon M, Fau G, Née G, Ehtisham J, Morello R, Hamon M. Meta-analysis of the diagnostic performance of stress perfusion cardiovascular magnetic resonance for detection of coronary artery disease. J Cardiovasc Magn Reson. 2010;12(1):29.
14. Khoo JP, Grundy BJ, Steadman CD, Sonnex EP, Coulden RA, McCann GP. Stress cardiovascular MR in routine clinical practice: referral patterns, accuracy, tolerance, safety and incidental findings. Br J Radiol. 2012;85(1018):e851-7.

Figure 1

Click here to access/download;Figure;Figure 1.tiff

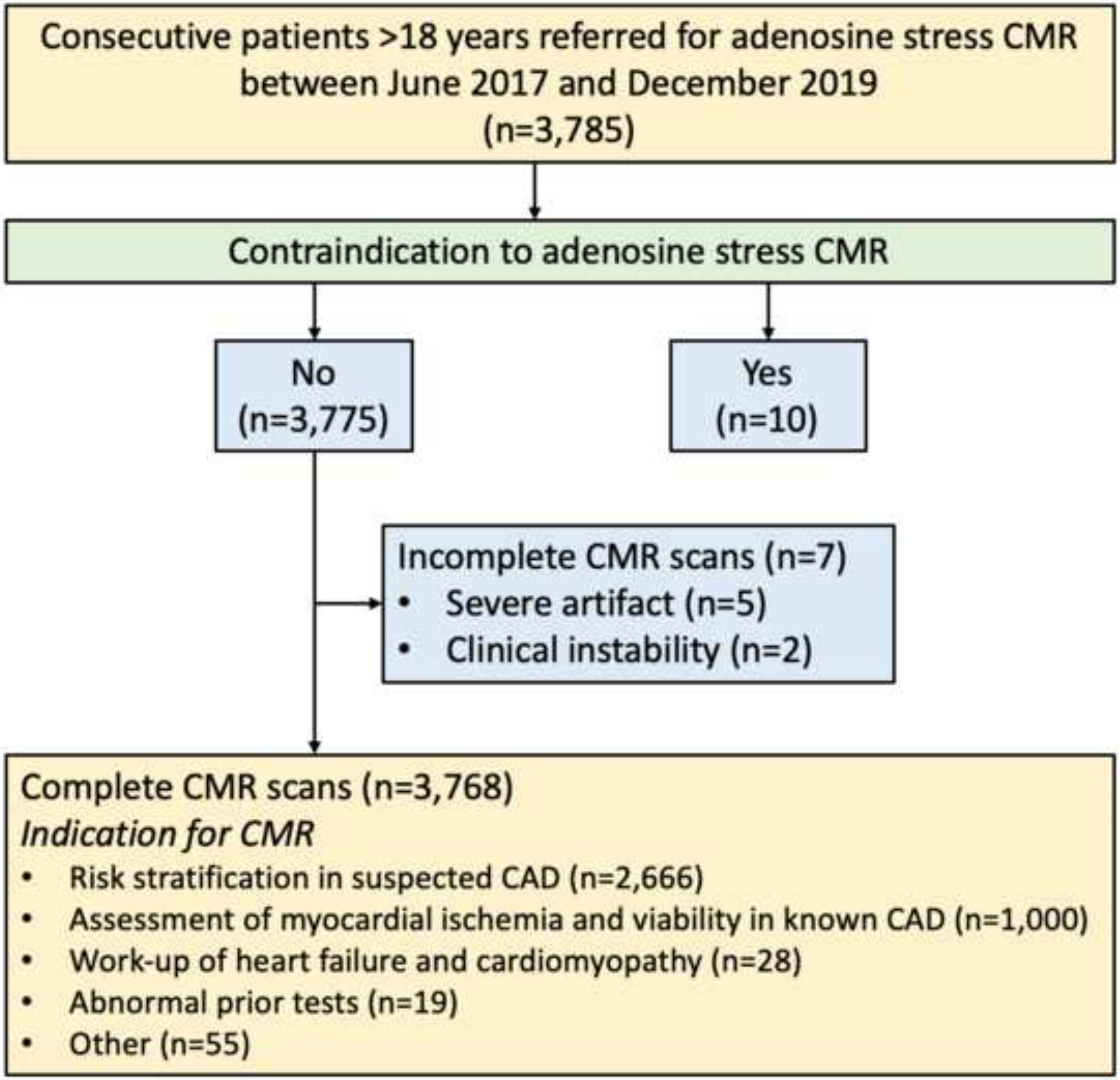

Figure 2

Click here to access/download;Figure;Figure 2.tiff

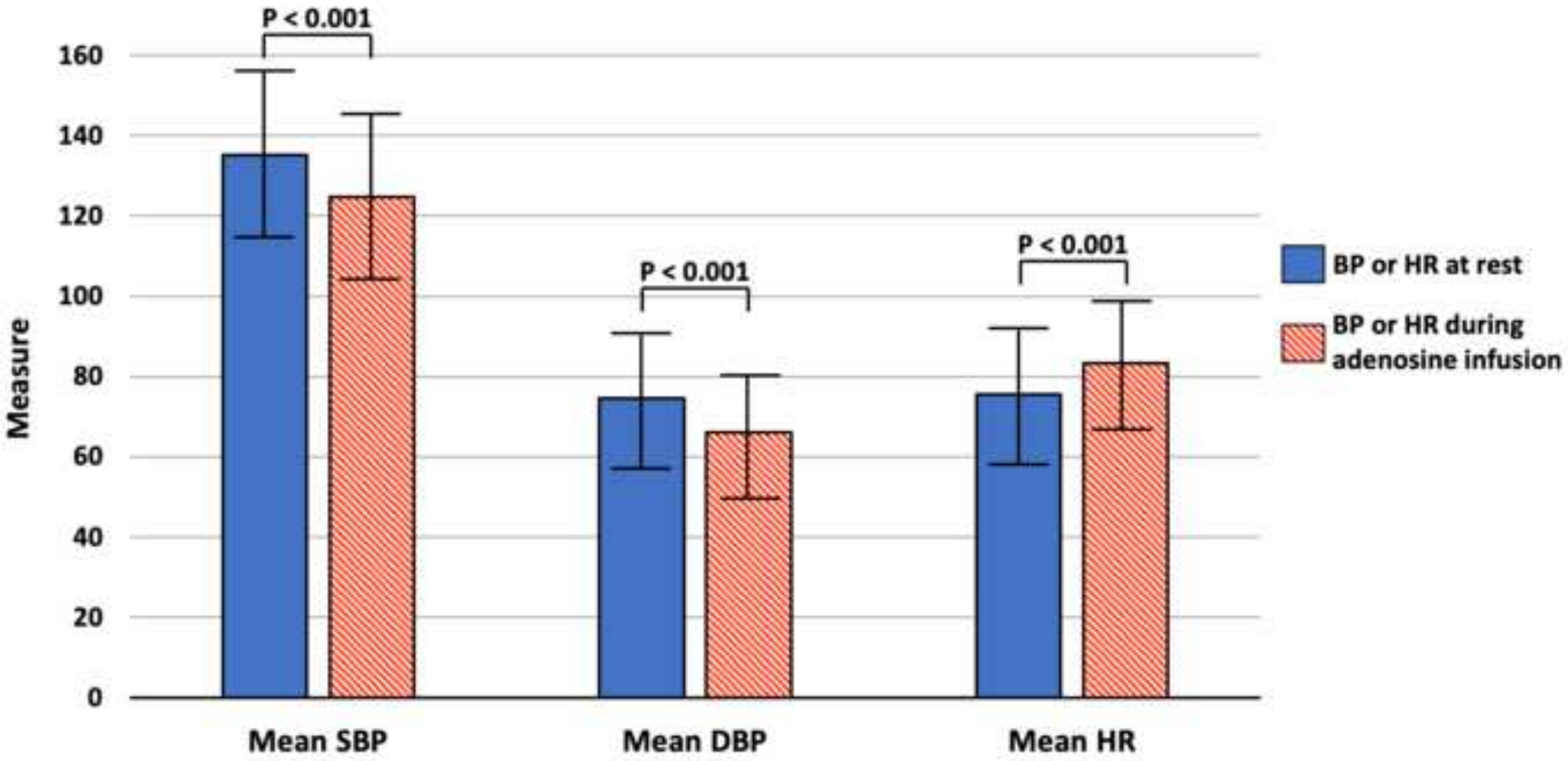

Figure 3

Click here to access/download;Figure;Figure 3.tiff

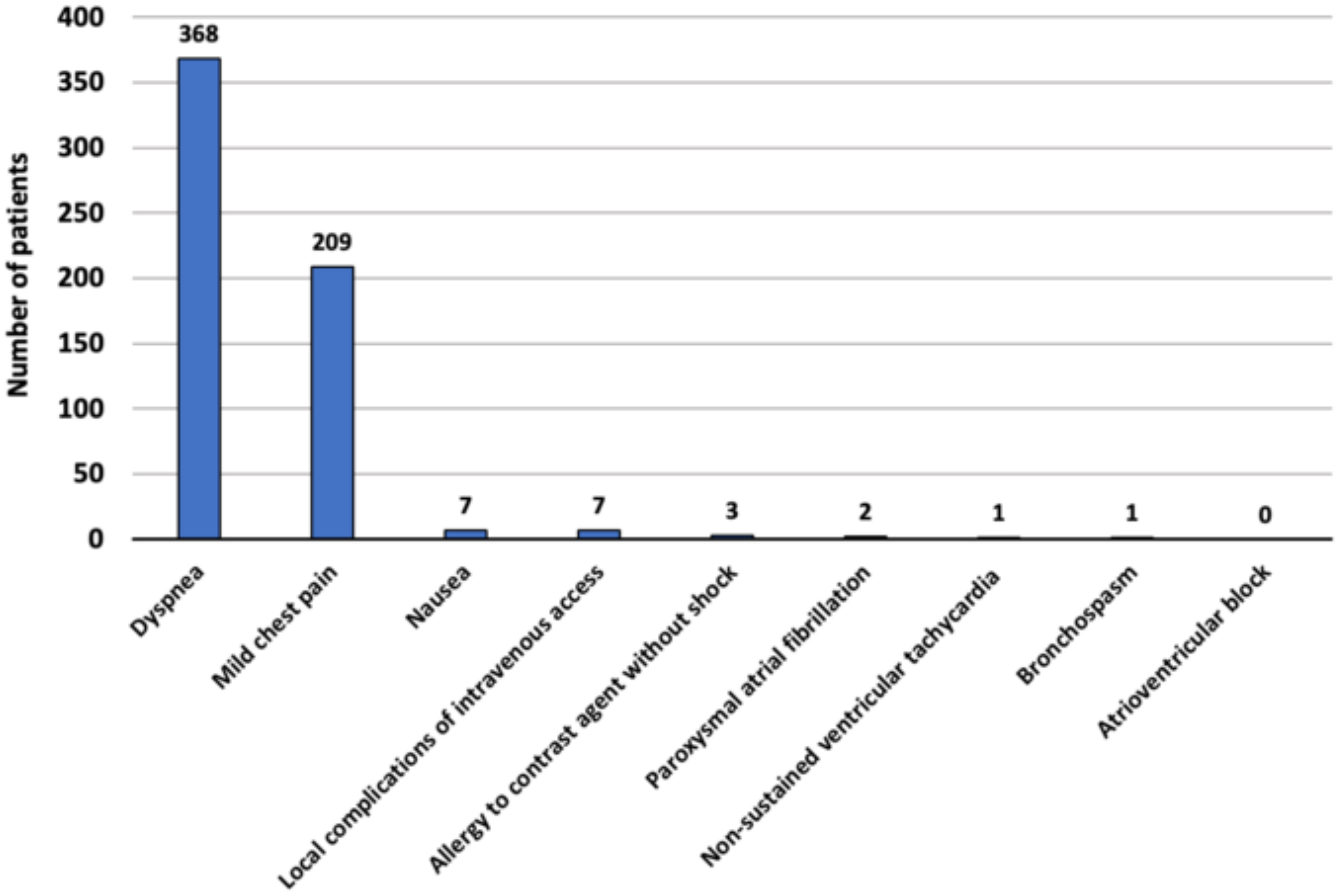

Supplement: Attachment — Submitted filename: PONE-D-23-23709_reviewer.pdf [file pone.0292950.s001.pdf]
